# Chilling injury of tomato fruit was alleviated under low-temperature storage by silencing Sly-miR171e with short tandem target mimic technology

**DOI:** 10.3389/fnut.2022.906227

**Published:** 2022-07-25

**Authors:** Keyan Zhao, Rulong Chen, Wenhui Duan, Lanhuan Meng, Hongmiao Song, Qing Wang, Jiangkuo Li, Xiangbin Xu

**Affiliations:** ^1^School of Food Science and Engineering, Hainan University, Haikou, China; ^2^Beijing Academy of Agriculture and Forestry Sciences, Beijing, China; ^3^Tianjin Key Laboratory of Postharvest Physiology and Storage of Agricultural Products, National Engineering and Technology Research Center for Preservation of Agricultural Products, Tianjin, China; ^4^Key Laboratory of Food Nutrition and Functional Food of Hainan Province, Haikou, China

**Keywords:** microRNA, tomato fruit, chilling injury, GRAS, GA

## Abstract

In this study, the role of Sly-miR171e on post-harvest cold tolerance of tomato fruit was researched. The results showed that overexpression of Sly-miR171e (miR171e-OE) promoted postharvest chilling injury (CI) of tomato fruit at the mature red (MR) and mature green (MG) stage. Contrasted with the wild type (WT) and miR171e-OE fruit, the knockdown of Sly-miR171e (miR171e-STTM) showed a lower CI index, lower hydrogen peroxide (H_2_O_2_) content, and higher fruit firmness after harvest. In the fruit of miR171e-STTM, the expression level of *GRAS24, CBF1, GA2ox1*, and *COR*, and the GA3 content were ascended, while the expression levels of *GA20ox1* and *GA3ox1* were descended. The research demonstrated that CI in tomato fruit was alleviated at low temperature storage by silencing Sly-miR171e with short tandem target mimic (STTM) technology. Furthermore, it also provided helpful information for genetic modification of miR171e and control of CI in the postharvest fruit.

## Introduction

Storage at low temperature is an effective method for controlling the quality of postharvest fruit, however, the chilling injury (CI) caused by low temperature often leads to a decline in fruit quality. The tomato fruit at the mature red (MR) and mature green (MG) stage is prone to CI when stored at temperature lower than 5 and 10°C, respectively. Tomato fruit stored at a low temperature usually shows symptoms like surface damage, rot, and tepbib1. In addition, low temperature affects the conformation and structure of the cell membranes of fruit, reduces consumer acceptability, and finally leads to significant economic losses (2, 3). So, improving the cold tolerance of postharvest fruit is a research hotspot in genetics and breeding.

MicroRNA (miRNA), which comprises 18–25 nucleotides, is a kind of endogenous single-stranded RNA. It plays a significant role in plant growth and development, resistance to stress (low temperature, drought, and salt stress), and maintenance of genomic integrity (4–8). In *Arabidopsis thaliana*, northern blot analysis showed that the overexpression of miR397 affected the expression of the cold-regulated *CBF* and *COR* genes, enhanced the cold tolerance in plants, and indicated that miR397 was involved in cold resistance (4). The results of small RNA sequencing and expression analysis in cold-treated grapevine plants showed that miR395 was significantly up-regulated, indicating that miRNA might be involved in cold stress in plants (5). Under low-temperature storage, compared with WT fruit, silencing of miR164a reduced the CI index and H_2_O_2_ content of tomato and improved the cold tolerance of fruit, indicating that miRNA could respond to cold stress (6). The miR528 improved the cold resistance of the rice by curbing the expression level of *MYB30* and increasing ascorbate peroxidase (9). During low temperature storage, overexpression of miR319 in tomatoes reduced the electrolyte leakage and malondialdehyde (MDA) content and played a role in cold resistance (10). It was demonstrated that miR164 played a role in regulating NAC expression in strawberry fruit stored at low temperatures (7, 8).

miR171 is one of the conservative miRNA families in plants and participates in the development and stress responses by down-regulating the expression of GRAS family genes (9, 10). In *A. thaliana*, protein localization and yeast one-hybrid analysis showed that miR171c negatively regulated the gene family members of *GRAS, SCL6-II, SCL6-III*, and *SCL6-IV*, and caused the bud-reducing branching phenotype in mutant plants, indicated that miR171c plays a vital role in plant development (11). In the peach tree (*Prunus persica* L.), quantitative real-time PCR (qRT-PCR) results showed that miR171 was highly expressed under drought and salt stress, indicating that it played an essential role in resisting abiotic stress (12). High-throughput analysis results showed that miR171 was highly expressed in celery plants under drought, indicating that it was responsive to drought stress (13). GRAS is one of the transcriptional regulators induced in plants to adapt to cold stress. As an important family of plant-specific proteins, GRAS is usually present in the C-terminal part of the protein and plays important roles in plant development and abiotic stresses (14–16). In *A. thaliana*, the DELLA protein of the GRAS subfamily reduced hydrogen peroxide (H_2_O_2_) accumulation and enhanced stress resistance by increasing the expression of genes encoding active oxygen detoxification enzymes (ROS) (17). In tomato fruit, *SlGRAS4* showed a significant increase under low temperature stress, and the overexpression of *SlGRAS4* increased the cold tolerance (15, 18).

In tomatoes, *SlGRAS24* is the target gene of miR171 (15, 19, 20). So far, there was no report about the role of miR171e on the cold tolerance of fruit by negatively regulating the target gene of *GRAS*. In the present research, silence and overexpression of Sly-miR171e tomato fruit were gained, and the function of Sly-miR171e on low temperature stress in postharvest tomato fruit was explored. The results showed that silencing Sly-miR171e with a short tandem target mimic (STTM) in tomato fruit led to less CI at low temperature storage.

## Materials and methods

### The construction of plant vector

The STTM structure of miR171e was constructed according to the method of Yan et al. (21) (Supplementary Material 1). The miR171e overexpression vector was constructed by the method of Qin et al. (22) (Supplementary Material 2). The target fragment of miR171e was obtained from miRbase and transferred into pBWA (V) HS and pBI121 vectors containing CaMV35S promoter. The sequences of candidate vectors were identified and then the vectors were introduced into *Agrobacterium* GV3101. The primers that appeared in the experiment are listed in Supplementary Material 3.

### Fruit and treatment

The tomato fruit (*Solanum lycopersicum cv*. Micro-Tom) at MR and MG stage without physical damage and infection was selected, put into the foam box, and then classified according to the size. The fruit at the MR stage was stored at 4°C, and the fruit at the MG stage was stored at 9°C. During the period of low temperature storage, three fruit were extracted from each group for 0, 5, 10, 15, 20, and 25 days, and the middle part of tomato fruit was selected for sampling.

### H_2_O_2_ content and CI index

The CI index of tomato fruit was determined by the method of Zhao et al. (23), in which 0 = no pitting; 1 = the depression covers < 25% of the fruit surface; 2 = pitting coverage > 25% and < 50% of the surface; 3 = pitting coverage >50% and < 75% of the surface, 4 = pitting coverage > 75% Surface. Calculate using the following formula:


$$\begin{array}{l} \text{CIindex}=\frac{\Sigma \left(\text{CI level}\right)\times \;\left(\text{number of fruit at the CI level}\right)}{\text{total number of fruit}}\;\times \;4. \end{array}$$

The H_2_O_2_ content was measured by the analytical kit (Solarbio Inc Beijing, China), and the results of the test were expressed as μ mol kg^−1^ fresh weight (FW).

### Electrolyte leakage and firmness of tomato fruit

The electrolyte leakage was measured according to Zhao et al. (24). The peel was separated using a 0.5 cm diameter separator, placed in the solution, and shaken on a shaker for 2 h, measuring the conductivity L1 using a conductivity meter (FE30), boiling for 10 min, cooling, and finally determining L2. Ion leakage was calculated as the ratio of L1 to L2.

The equatorial position in the fruit was measured three times with texture analysis (TA. XT Plus). The insertion speed was 50 mm s^−1^, the insertion depth was 5 mm, and the results of the test were expressed as N.

### The measure of GA3 content

The GA3 content was measured with the detection kit (Jiangsu Meibiao Biotechnology Co., Ltd.). The result was expressed as mg L^−1^.

### qRT-PCR analysis and RNA extraction

The total RNA of WT, miR171e-STTM, and miR171e-OE tomato fruit was extracted by using the plant RNA extraction kit (Tiangen Biotech Inc. Beijing, China), respectively. And then the concentration and quality of RNA were determined. The first-strand cDNA was synthesized using the FastKing RT kit (Tiangen Biotech Inc. Beijing, China), and the expression of Sly-miR171e, *GRAS24, GA3ox1, GA20ox1, GA2ox1, GAI, CBF1*, and *COR* was analyzed by the CFX Connect Real-Time System (Bio-Rad Laboratories, Inc. USA). The expression level was calculated using the formula: 2^−ΔΔCt^.

### Statistical analysis

GraphPad Prism 9 (GraphPad, San Diego, CA, USA) and a two-way analysis of variance (ANOVA) were used for statistical analysis. ^*^p < 0.05 indicate significant differences in comparison with wild type (WT). All values were expressed as the average value ± standard deviation (SD).

## Results

### Characterization of MiR171e-STTM and MiR171e-OE fruit

The STTM and overexpression vectors were introduced into WT to obtain the miR171e-STTM and miR171e-OE lines through genetic transformation, which can express stably. In the first generation (T1), 23 STTM-171e lines and 17 miR171e-OE lines were tested (Supplementary Figures S1A,B). In the second generation (T2), 16 STTM-171e lines and 17 miR171e-OE lines were tested (Supplementary Figures S1C,D). The results of PCR amplification and agarose gel electrophoresis showed that both STTM and overexpression vectors had been inserted into the correct target sites (Supplementary Figure S1).

### H_2_O_2_ content and CI index

**Figure 1:**
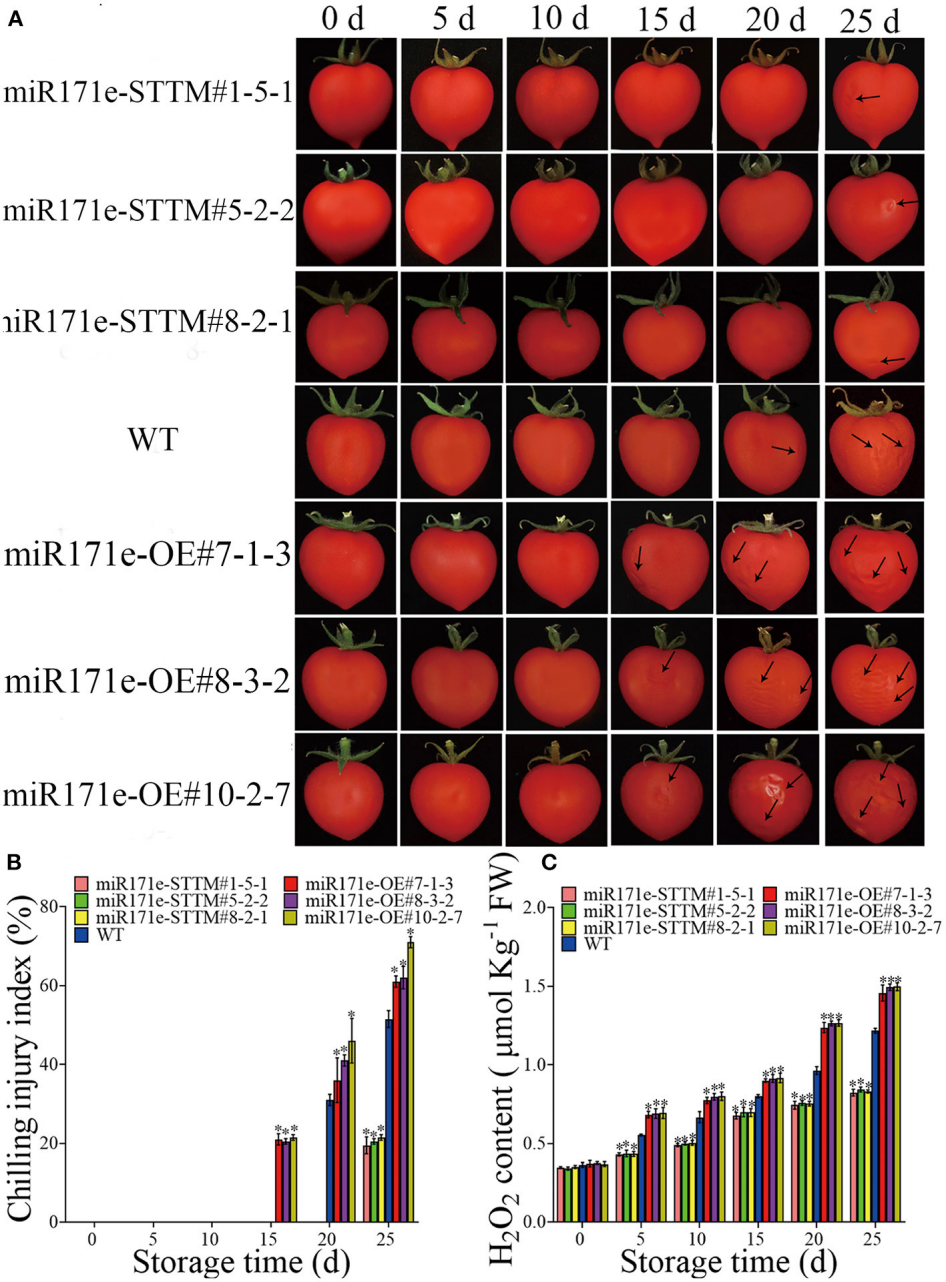
The CI and phenotypic characterization in tomato fruit at MR stage. **(A)** The H_2_O_2_ content of tomato fruit at MR stage. **(B)** The CI index of tomato fruit at MR stage. **(C)** Vertical bars represent standard deviations of the means, *n* = 3. Asterisks indicate the statistical difference in the significance at *p < 0.05.

Figures 1A,B showed that the fruit of miR171e-OE#7-1-3, miR171e-OE#8-3-2, and miR171e-OE#10-2-7 at the MR stage were shrunken at 15 d and showed obvious CI at 25 d. After 20 d at low temperature, CI appeared in WT fruit at the MR stage. However, the fruit of miR171e-STTM#1-5-1, miR171e-STTM#5-2-2, and miR171e-STTM#8-2-1 at the MR stage showed less cold damage after 25 d of storage. In addition, during low temperature storage of 4°C, the H_2_O_2_ content of the miR171e-STTM fruit was often less than that in WT (Figure 1C).

**Figure 2:**
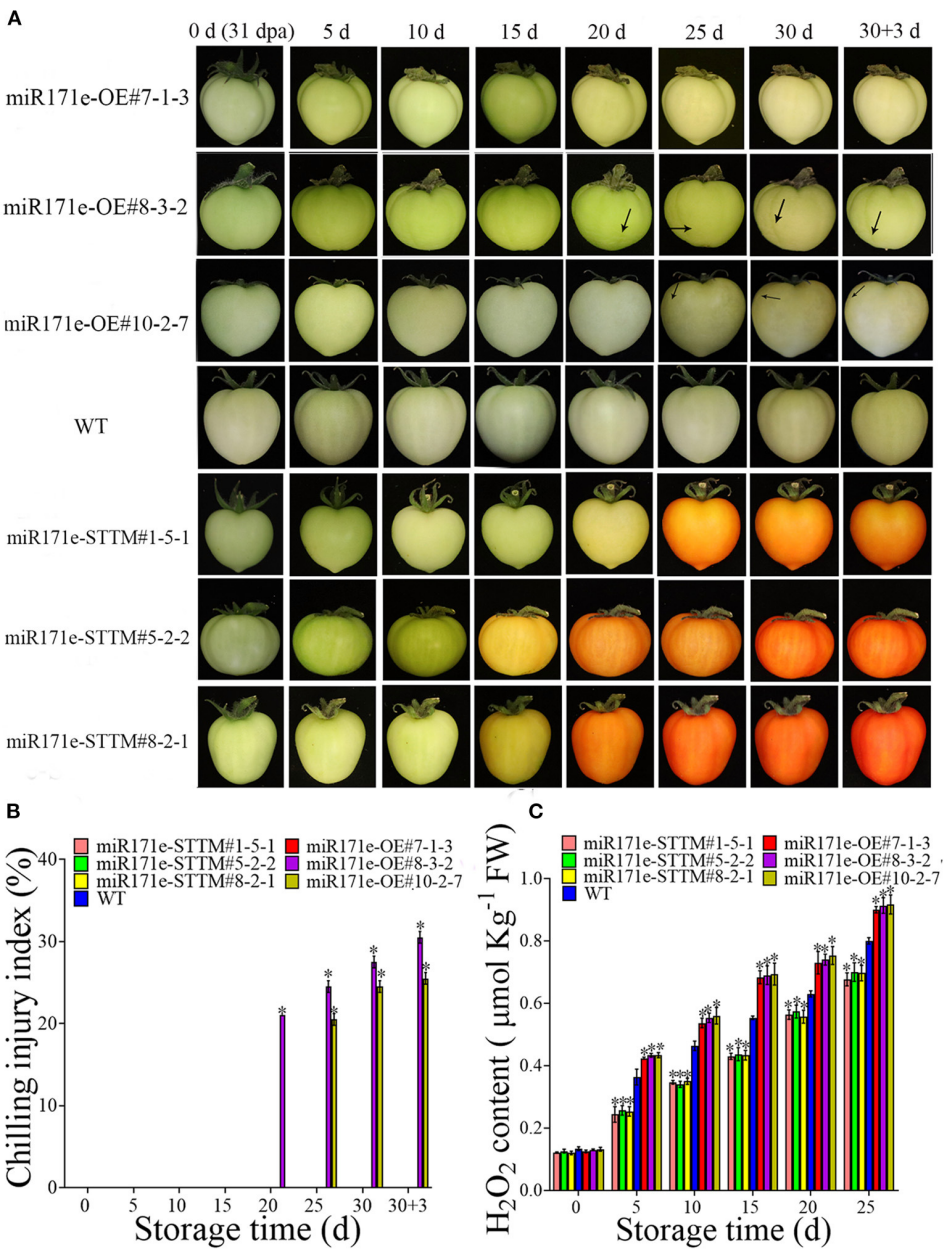
The CI and phenotypic characterization in tomato fruit at MG stage. **(A)** The H_2_O_2_ content of tomato fruit at MG stage. **(B)** The CI index of tomato fruit at MG stage. **(C)**. Vertical bars represent standard deviations of the means, *n* = 3. Asterisks indicate the statistical difference in the significance at *p < 0.05.

As shown in Figures 2A,B, after 20 days of storage, the tomato fruit of miR171e-OE#8-3-2 at the MG stage was shrunken and exhibited CI, and the miR171e-OE#10-2-7 line showed CI at 25 d. However, after being stored at 9°C for 30 d and the fruit was transferred to 25°C, the fruit of WT and miR171e-STTM at the MG stage showed no cold damage. The H_2_O_2_ content in miR171e-STTM#1-5-1, miR171e-STTM#5-2-2, and miR171e-STTM#8-2-1 fruit at MG stage was 0.82, 0.85 and 0.84 times less than that in WT at 25 days, respectively (Figure 2C).

### Electrolyte leakage and firmness of tomato fruit

**Figure 3:**
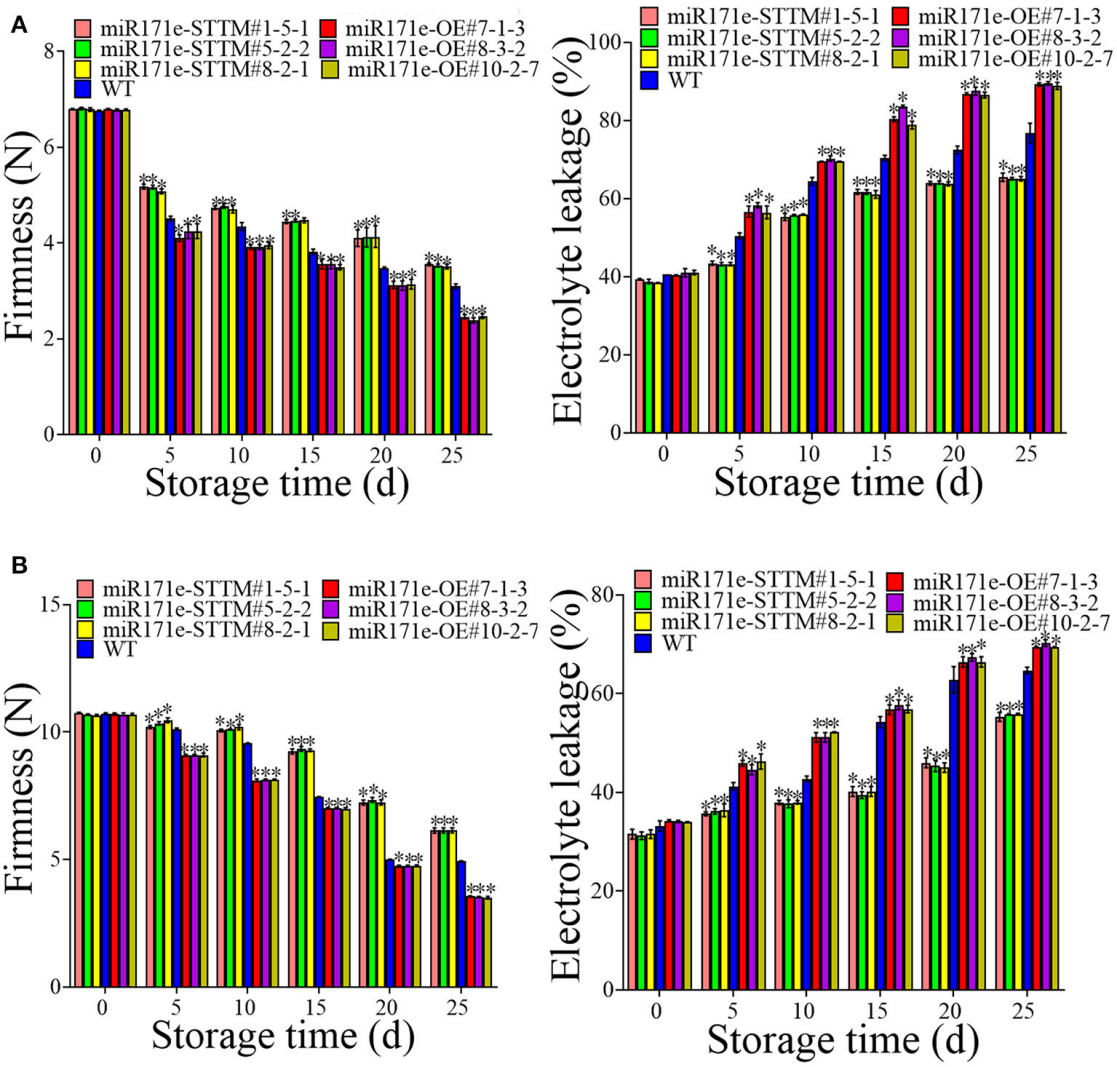
Electrolyte leakage and firmness in tomato fruit at MR **(A)** and MG stage **(B)**. Vertical bars represent standard deviations of the means, *n* = 3. Asterisks indicate the statistical difference in the significance at *p < 0.05.

In contrast to WT fruit at the MR stage, the electrolyte leakage was reduced in the miR171e-STTM and was increased in miR171e-OE (Figure 3A). After 5 d of storage, the electrolyte leakage in fruit of miR171e-STTM#1-5-1, miR171e-STTM#5-2-2, miR171e-STTM#8-2-1, miR171e-OE#7-1-3, miR171e-OE#8-3-2 and miR171e-OE#10-2-7 were 43, 42, 43, 56, 57, and 56%, respectively (Figure 3A). As shown in Figure 3A, after 25 days of storage, electrolyte leakage in WT fruit at the MR stage was 70%, which was 1.18, 1.2, and 1.18 times more than that in miR171e-STTM#1-5-1, miR171e-STTM#5-2-2, and miR171e-STTM#8-2-1, respectively. Contrary to the WT fruit at the MR stage, the firmness of miR171e-STTM fruit was increased, and the firmness of the miR171e-OE fruit was reduced. After storing for 25 days at low temperature, the firmness of WT fruit was 3.09 N, which was 1.15, 1.14, and 1.12 times more than that of miR171e-STTM#1-5-1, miR171e-STTM#5-2-2, and miR171e-STTM#8-2-1, respectively (Figure 3A).

As shown in Figure 3B, in contrast with WT fruit, the electrolyte leakage in miR171e-STTM tomato fruit at the MG stage was reduced and increased in miR171e-OE fruit. After 5 days of storage, the electrolyte leakage of WT fruit was 41%, which was 1.17, 1.13, and 1.12 times more than that in miR171e-STTM#1-5-1, miR171e-STTM#5-2-2, and miR171e-STTM#8-2-1 of the tomato fruit at MG stage, respectively. The electrolyte leakage in WT fruit was 65%, which was 1.18, 1.16, and 1.16 times more than that in miR171e-STTM#1-5-1, miR171e-STTM#5-2-2, and miR171e-STTM#8-2-1 at 25 days, respectively. As shown in Figure 3B, after being stored for 0 to 25 days at low temperature, the firmness of miR171e-STTM fruit diminished from 10.69 to 6.14 N, which was more than that of WT fruit.

### The GA3 content and related genes expression

**Figure 4:**
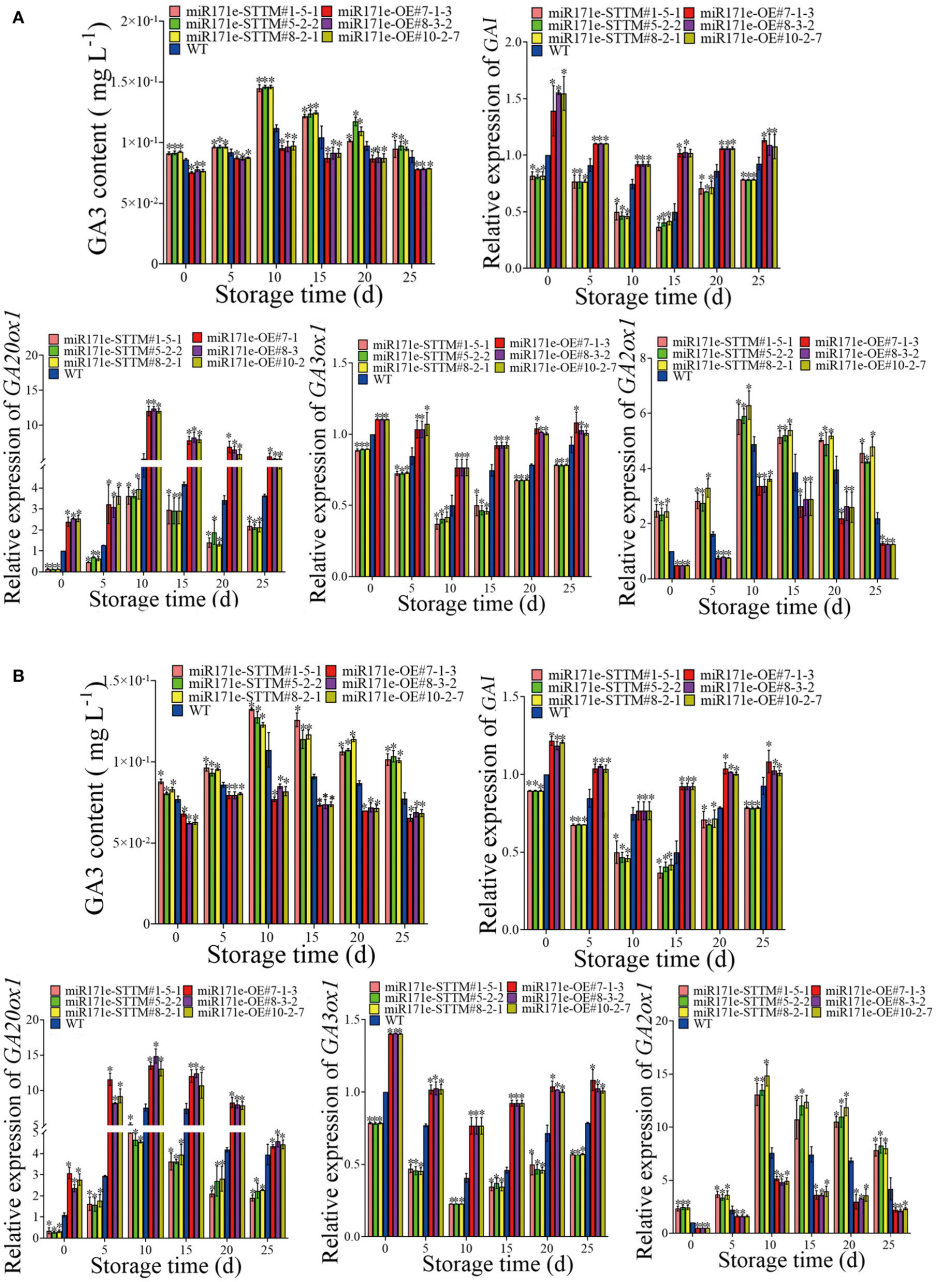
The GA3 content and the related gene expression in tomato fruit at MR **(A)** and MG stage **(B)**. Vertical bars represent standard deviations of the means, *n* = 3. Asterisks indicate the statistical difference in the significance at *p < 0.05.

During storage from 0 to 25 days, contrary to the WT fruit at the MR stage, the GA3 content of the miR171e-STTM fruit was increase, and the GA3 content of the miR171e-OE fruit was reduced. The GA3 content was 0.0885 mg L^−1^ in WT fruit, which was 0.93, 0.9, and 0.93 times less than that in miR171e-STTM#1-5-1, miR171e-STTM#5-2-2, and miR171e-STTM#8-2-1 fruit at 25 days, respectively (Figure 4A). As shown in Figure 4A, the expression level of the DELLA protein gene (*GAI*) in miR171e-STTM#1-5-1, miR171e-STTM#5-2-2, and miR171e-STTM#8-2-1 fruit at MR stage was about 0.82, 0.87, and 0.83 times less than that in WT at 25 days. Moreover, the expression level of the critical GA synthesis genes *GA3ox1* and *GA20x1* in miR171e-STTM tomato fruit at the MR stage was about 0.78 and 0.61 times less than that in WT (Figure 4A). As shown in Figure 4A, at 25 days, the expression level of *GA2ox1*, which was the critical gene of GA metabolism of miR171e-STTM fruit at the MR stage, was about 2.06 times of WT fruit.

As shown in Figure 4B, contrary to the WT fruit at the MG stage, the GA3 content of the miR171e-STTM was increased, and the miR171e-OE was reduced. After 25 days of storage, the GA3 content in WT fruit was 0.077 mg L^−1^, which was 0.76, 0.74, and 0.76 times less than that in miR171e-STTM#1-5-1, miR171e-STTM#5-2-2, and miR171e-STTM#8-2-1 fruit, respectively. The expression level of the *GAI* in miR171e-STTM#1-5-1, miR171e-STTM#5-2-2, and miR171e-STTM#8-2-1 tomato fruit at the MG stage was about 0.84, 0.85, and 0.85 times less than that in WT at 25 days. After 25 days at low temperature storage, the expression level of *GA3ox1* and *GA20x1* in the critical GA synthesis genes of miR171e-STTM fruit at the MG stage was about 0.70 and 0.56 times less than that in WT (Figure 4B), and the expression level of *GA2ox1* was about 1.97 times of WT (Figure 4B).

### Expression level of MiR171e and *GRAS24*

**Figure 5:**
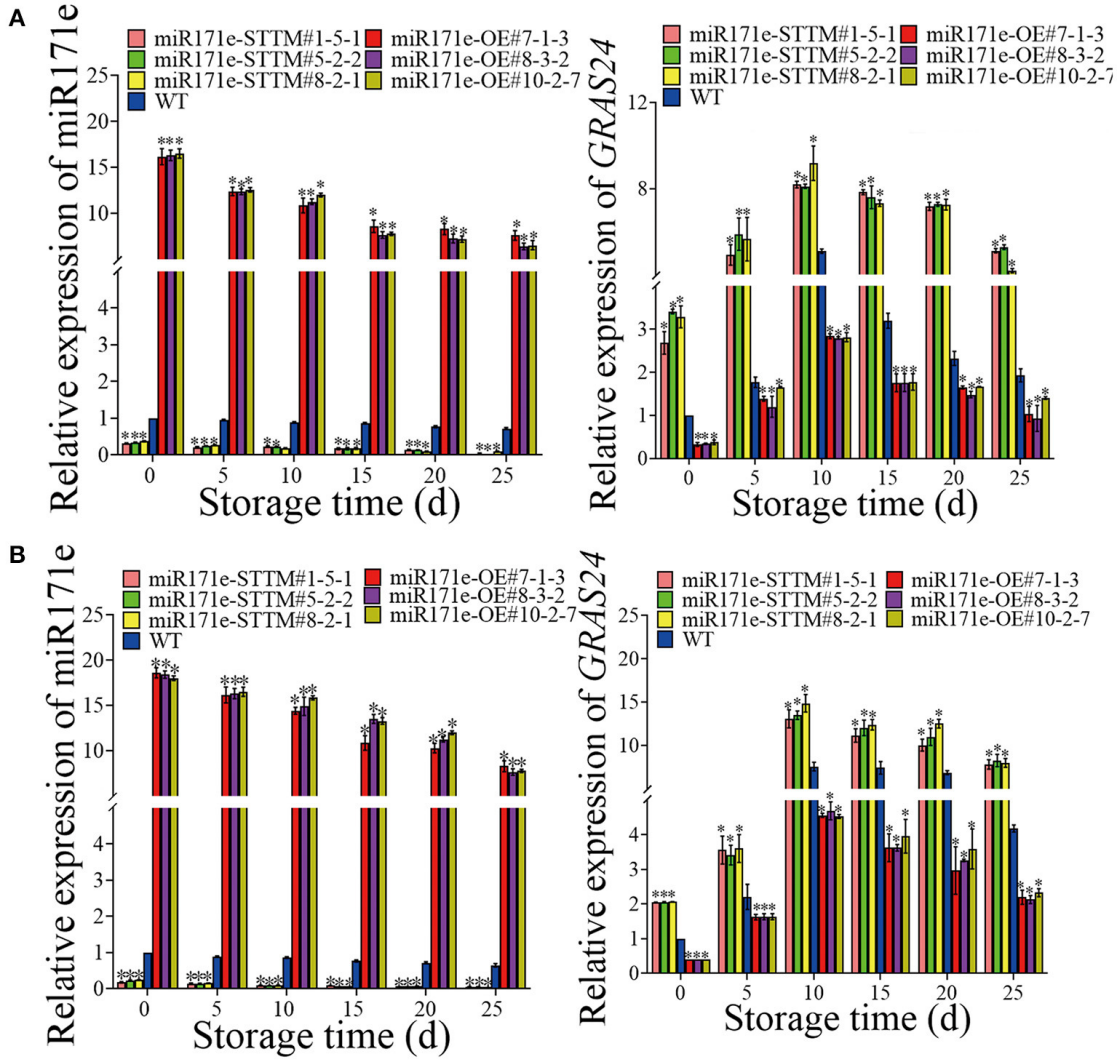
The relative expression of Sly-miR171e, *GRAS24* in tomato fruit at MR **(A)** and MG stage **(B)**. Vertical bars represent standard deviations of the means, *n* = 3. Asterisks indicate the statistical difference in the significance at *p < 0.05.

As shown in Figure 5A, after 25 days of storage, the expression level of Sly-miR171e of miR171e-STTM#1-5-1, miR171e-STTM#5-2-2, and miR171e-STTM#8-2-1 fruit at MR stage was about 0.53, 0.27, and 0.14 times less than that in WT. Contrasted with WT fruit at the MR stage, the expression level of *GRAS24* in miR171e-STTM fruit was increased and decreased in the miR171e-OE fruit. After 25 days at low temperature, the expression of *GRAS24* in WT fruit was 1.94, which was 2.64, 2.7, and 2.27 times more than that in miR171e-STTM#1-5-1, miR171e-STTM#5-2-2, and miR171e-STTM#8-2-1 fruit, respectively (Figure 5A).

The expression level of Sly-miR171e in miR171e-STTM#1-5-1, miR171e-STTM#5-2-2, and miR171e-STTM#8-2-1 fruit at MG stage was about 0.15, 0.13 and 0.14 times less than that in WT at 25 d (Figure 5B). After 25 days of storage, the expression level of *GRAS24* in WT fruit was 4.18, which was 1.9, 2.0, and 2.0 times more than that in miR171e-STTM#1-5-1, miR171e-STTM#5-2-2, and miR171e-STTM#8-2-1 fruit, respectively (Figure 5B).

### Expression level of *CBF1* and *COR*

**Figure 6:**
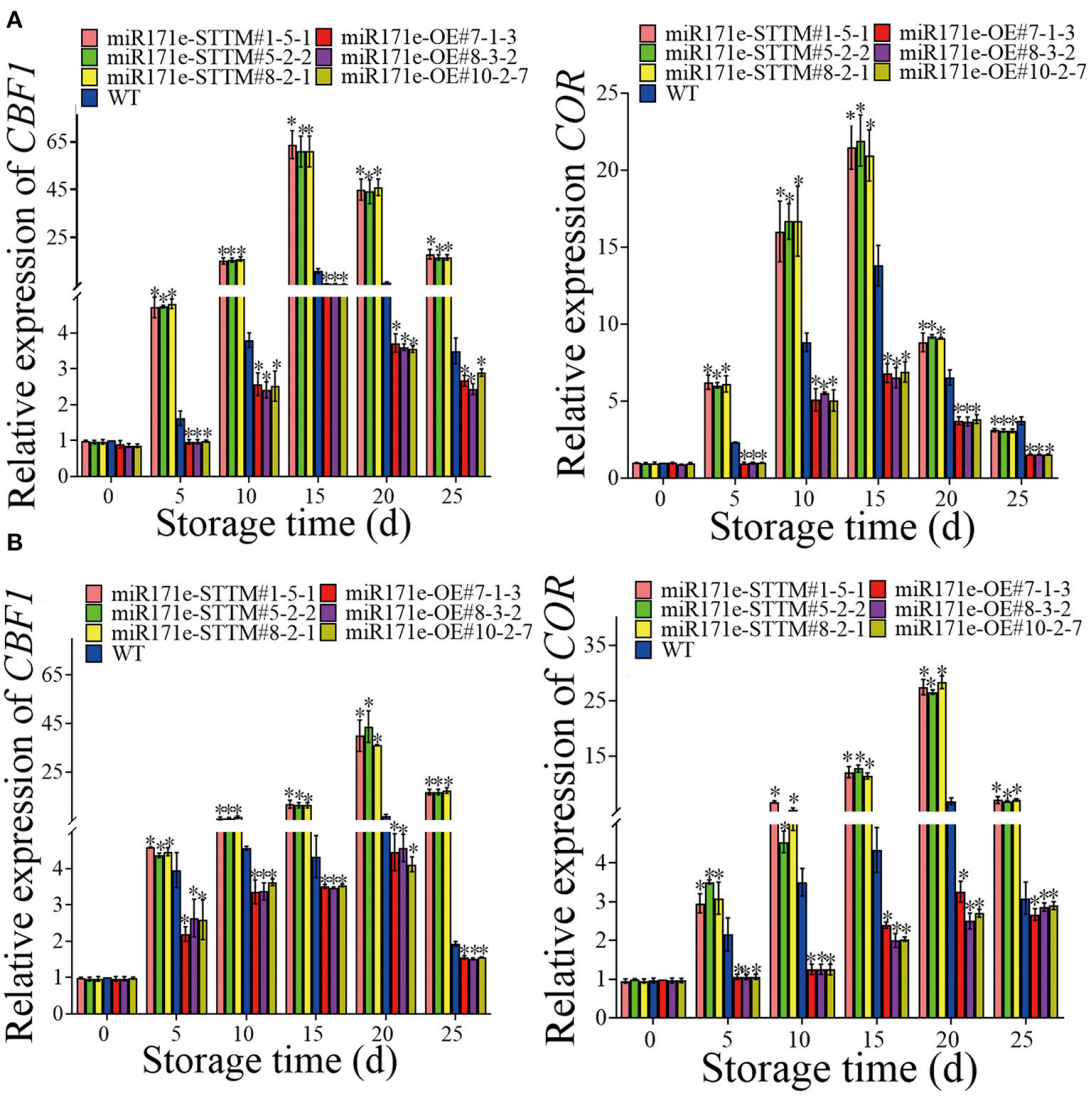
The relative expression of *CBF1* and *COR* in tomato fruit at MR **(A)** and MG stage **(B)**. Vertical bars represent standard deviations of the means, *n* = 3. Asterisks indicate the statistical difference in the significance at *p < 0.05.

As shown in Figure 6A, during storage from 0 to 25 d, contrasted with WT fruit at the MR stage, the expressions of *CBF1* and *COR* in the miR171e-STTM fruit were ascending and descending in the miR171e-OE fruit. After 25 d of storage, the expression level of *CBF1* of miR171e-STTM#1-5-1, miR171e-STTM#5-2-2, and miR171e-STTM#8-2-1 fruit was about 5.14, 4.85, and 4.85 times more than that in WT and the expression level of *COR* in miR171e-STTM#1-5-1, miR171e-STTM#5-2-2, and miR171e-STTM#8-2-1 fruit were about 1.46, 1.4, and 1.39 times more than that in WT (Figure 6A).

After 25 days of storage, the expression level of *CBF1* in miR171e-STTM#1-5-1, miR171e-STTM#5-2-2, and miR171e-STTM#8-2-1fruit at the MG stage was about 8.5, 8.5, and 8.6 times more than that in WT (Figure 6B), and the expression level of *COR* in miR171e-STTM#1-5-1, miR171e-STTM#5-2-2, and miR171e-STTM#8-2-1 fruit were about 2.29, 2.25, and 2.3 times more than that in WT (Figure 6B).

## Discussion

The structural damage of the cell membrane is the leading cause of CI in refrigerated fruit (2). At low temperature, the cell membrane structure changed between a liquid crystal and a gel state, accompanied by an increase in the membrane lipid unsaturated fatty acid content, which led to a rise in membrane permeability and ion leakage, and finally caused CI (25–27). In the CI study, compared with the WT and overexpression of Sly-miR171e, silencing Sly-miR171e improved the hardness of tomato fruit, reduced its electrolyte leakage, and improved cold tolerance in the fruit. At low temperatures, plants will produce oxidative stress, that is, excessive production of ROS, including H_2_O_2_ and superoxide anion (O^2−^) (28, 29). H_2_O_2_ is a second messenger of defense response (30–32) and is a substance that causes damage to cell membranes. Excessive accumulation of H_2_O_2_ under CI conditions results in degradation of membrane fatty acids and deepening of CI. In the present study, the H_2_O_2_ content of miR171e-OE fruit was induced and accumulated to the CI phenotype at 20 and 25 days of MG stage and at 15 days of MR stage, respectively. Compared with WT and miR171e-OE, the H_2_O_2_ content of miR171e-STTM tomato fruit decreased and showed less sensitivity to CI.

GA, an important plant hormone, is widely distributed in higher plants. It participates in the growth and development of plants, and also plays a vital role in the adaptability of plants at low temperatures (25, 33–35). Low temperature stress could up-regulate the expression of the *GA2ox* gene in *A. thaliana* seedlings, reduce the content of active GA *in vivo*, increase the content of the DELLA protein, and inhibit the growth of seedlings (25). Postharvest GA3 treatment could reduce fruit softening and cold damage during storage, and prolong their shelf life (35). The GA3 (0.5 mM) treatment could effectively maintain the stability of the tomato fruit membrane, maintain the integrity of the cell wall and reduce the activity of antioxidant enzymes under low temperature storage, thus alleviating the CI of postharvest tomato fruit (34). Under low temperature, GA3 treatment reduced CI, electrolyte leakage, and MDA content and alleviated the CI of the cherry tomato fruit (35). *GA20ox, GA3ox*, and *GA2ox* are essential regulators of GA participation in abiotic stress (36, 37). In *A. thaliana*, when the bioactivity of GA was at a low level, the expression of three *GA20ox* genes (*GA20ox1, GA20ox2*, and *GA20ox3*) and one *GA3ox1* gene (*GA3ox1*) is up-regulated; in contrast, the expression of *GA2oxs*, an enzyme related to GA inactivation, was down-regulated and resulted in GA biosynthesis and accumulation. However, after treatment with exogenous GA, the expression of these genes was just the opposite (38, 39). The DELLA protein is located in the nucleus and mainly inhibits plant growth and development by repressing gene transcription. Exogenous GA3 treatment can down-regulate the expression of the DELLA protein gene (*GAI)* of tomato fruit and abiotic stress resistance (33). In *A. thaliana*, the expression of GA synthase genes *GA20ox* and *GA3ox* was up-regulated when GA content was low (high DELLA protein content) (36). GA steady-state positive feedback regulates the *GA2ox* gene, and with the increase of GA signal output, the transcription level of *GA2ox* is up-regulated in the plant (40). In the present study, contrasted with the WT, the content of GA3 increased and the expression level of the *GAI* gene decreased in miR171e-STTM tomato fruit, which might inhibit the ripening and senescence of the fruit in cold storage and delay the CI of the fruit. In addition, contrasted with the WT, the expression levels of *GA20ox1* and *GA3ox1* decreased and the expression level of *GA2ox1* increased in miR171e-STTM fruit, which might be related to the improvement of postharvest cold tolerance in tomato fruit under low temperature storage.

**Figure 7:**
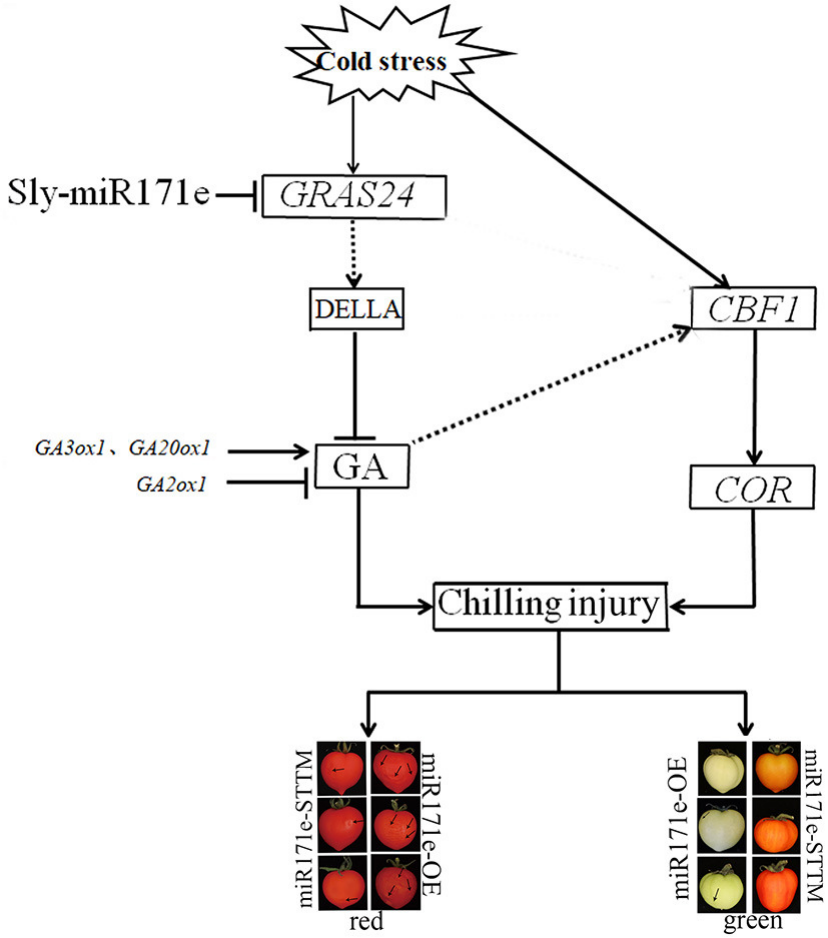
Regulation mechanism of Sly-miR171e on cold tolerance in tomato fruit stored at low temperature.

*CBF* gene plays an essential role in plant cold response (23, 41). Cold tolerance of plants was increased by overexpressing *CBF2, CBF3*, and *CBF1*, and then CBF transcription factors led to the expression of the *COR* gene (42). Cold stress can only effectively induce the *CBF1* gene in tomatoes, and the expression of *CBF* could increase rapidly and reach the maximum under transient cold stress treatment (43, 44). The correlation between the expression level of *CBF1* and the cold resistance of tomato can be used to measure the cold resistance of tomato fruit (23). Plant hormone assays and qRT-PCR analyses showed that overexpression of *CBF1* in *A. thaliana* induced the expression of the *GA2ox* and GA metabolism in response to low temperature (45). Results of microarray analysis and qRT-PCR showed that the expression levels of *CBF1* and *GA2ox1* in tobacco plants were consistent under low-temperature stress, indicating that *CBF1* and GA signals have a synergistic response at low temperature (46, 47). In the present results, the expression of *CBF1* was the same as that of *GA2ox1*. Therefore, we believed that the *CBF1* gene might have participated in the GA-induced cold resistance by regulating the expression of the *GA2ox1*. Expression analysis showed that higher GA content in overexpressed *GRAS24* than that in the control group, indicating that overexpression of *GRAS24* affected various agronomic traits by regulating GA (16). In the present study, Sly-miR171e might regulate the expression of *GRAS24*, which influenced the GA signaling and CBF pathway and improved the cold tolerance of postharvest fruit (Figure 7).

## Conclusion

The silencing of Sly-miR171e enhanced the expression of *GRAS24*, increased the GA content and the expression of *CBF1* and *COR*, and by which CI of tomato fruit was alleviated.

## Data availability statement

The original contributions presented in the study are included in the article/Supplementary materials, further inquiries can be directed to the corresponding author.

## Author contributions

KZ: writing—original draft. RC, WD, QW, HS, LM, and JL: availability of resources. KZ and XX: analysis of data. XX: writing—the revision of the manuscript. HS: funding. All authors have edited and approved the manuscript.

## Funding

This study was supported by the National Natural Science Foundation of China (31872160) and the Hainan Provincial Natural Science Foundation of China (321RC1025 and 2019RC127).

## Conflict of interest

The authors declare that the research was conducted in the absence of any commercial or financial relationships that could be construed as a potential conflict of interest.

## Publisher's note

All claims expressed in this article are solely those of the authors and do not necessarily represent those of their affiliated organizations, or those of the publisher, the editors and the reviewers. Any product that may be evaluated in this article, or claim that may be made by its manufacturer, is not guaranteed or endorsed by the publisher.
