# Supplementary material for: Chilling injury of tomato fruit was alleviated under low-temperature storage by silencing Sly-miR171e with short tandem target mimic technology
Source: Front Nutr. 2022 Jul 25;9:906227. doi: 10.3389/fnut.2022.906227 (PMC9355414; doi:10.3389/fnut.2022.906227)
Supplement: Supplementary file 1 [file Data_Sheet_1.docx]

The sequences of miR171e-STTM. Yellow shades indicate the 48 nt spacer. Green shades indicate the miR171e-STTM sequences. Blue shades and Bold indicate the *BsaI* and *Eco31I* enzyme digestion sites*,* respectively. Blue shades indicate the 2 ×35PS and T35PS*,* respectively.

>miR171e-STTM

CTTCAAAGCAAGTGGATTGATGTGATATCTCCACTGACGTAAGGGATGACGCACAATCCCACTATCCTTCGCAAGACCCTTCCTCTATATAAGGAAGTTCATTTCATTTGGAGAGAACACGGGGGACTTTGAGGCAACAACTCGGCGCTACAGTTATAGAGAGTTGTTGTTGTTATGGTCTAATTTAAATATGGTCTAAAGAAGAAGAATAACTCGGCGCTACAGTTATAGAGATGTAACTAGCTCTGTCTTCAGTACTGGGCCCGAAGACTGACCAGCTCGAATTCCCCGATCGTCAAACATTGCAATA AGTA
