# Supplementary material for: Chilling injury of tomato fruit was alleviated under low-temperature storage by silencing Sly-miR171e with short tandem target mimic technology
Source: Front Nutr. 2022 Jul 25;9:906227. doi: 10.3389/fnut.2022.906227 (PMC9355414; doi:10.3389/fnut.2022.906227)
Supplement: Supplementary file 2 [file Data_Sheet_2.docx]

The sequences of miR171e-OE. Yellow shades indicate the precursor sequence of miR171e. Green shades and bold indicate the mature miR171e sequences, respectively.

>miR171e-OE

GGTCACAACATGATGAAGATACATAGTTGAAACTTGAAAGATATAATAGT TTTGATTTTTCGTATTGAAAATTATTCTGCAAATTGATGGATAGCTAGCTAATTCAAAGA TGAAGTTGGG AAATATTGAA GGTACGTACT TTATAATATG TTTAGGAAGATATATATAGATATTGATGCGGTTCAATCTGAAAGACATGG TTAGATATGTAATTAGCCTTGTAATTTTGGA**TTGAGCCGCGTCAATATCTCT**CTTCCTATTTTCAATTAGTTTATAAGTAACTTGAACTTTATTTAATTACTCGTTGGTAATACTTGTCTTGTTTCATGTTTTCCTCTTGGCCATGCATCTTTAATGTTTTTTTTTCCACTAATTTTCTGGTTTTTAATTAGTTTTTAAATTTCTTCTTAATTTCATCTTTGACACCATTAATTTCATTCGTTGCCGTTCGTTGATAGAC GTTTTTGAATTAGATAG
