# Supplementary material for: Chilling injury of tomato fruit was alleviated under low-temperature storage by silencing Sly-miR171e with short tandem target mimic technology
Source: Front Nutr. 2022 Jul 25;9:906227. doi: 10.3389/fnut.2022.906227 (PMC9355414; doi:10.3389/fnut.2022.906227)
Supplement: Supplementary file 3 [file Data_Sheet_3.doc]

**Supplement 3.** Primers used in the present study for expression analyses of miRNAs and genes.

| Gene | Primer | Sequence (5^，^-3^，^) |
| --- | --- | --- |
| *SlGAI* | Forward Primer | AGGCAACAGTGAAACTTCCATCAAG |
|  | Reverse Primer | TCCATTAAAGAGGATATTACCGGGGAC |
| *SlGA20ox1* | Forward Primer | TGGTTTTCATGGGACATTCATTAGC |
|  | Reverse Primer | ATCTCCCTTCTCAACTCCCTATTCC |
| *SlGA3ox1* | Forward Primer | CCCAGAGTTCTTTCCTGATCCACAA |
|  | Reverse Primer | GAATGCCACTACCAGATCCTACCAC |
| *SlGA2ox1* | Forward Primer | TCGAAAAAGGATACAATTGCATGCC |
|  | Reverse Primer | CTGCAATTTGTTCGTCAGTGAGTCC |
| *TOM-U6* | Forward Primer | CGATAAAATTGGAACGATACAG |
|  | Reverse Primer | TTGGACCATTTCTCGATTTG |
| *SlGRAS24* | Forward Primer | GCTTGAGGTCTTGGATGG |
|  | Reverse Primer | TGTGGATGGTGAGGCTAA |
| *SlActin* | Forward Primer | CAGCAGATGTGGATCTCAAA |
|  | Reverse Primer | CTGTGGACAATGGAAGGAC |
| *CBF1* | Forward Primer | GGCAGAAGGACTTATGCTACC |
|  | Reverse Primer | ACCCAACAAGTTTCTGTTCATGT |
| *COR* | Forward Primer | GGCATCCGTTGAAGAGACTG |
|  | Reverse Primer | GGCATCCGTTGAAGAGACTG |
| RT-miR171e | Primer | GTCGTATCCAGTGCAGGGTCCGAGGTATTCGCACTGGATACGACAGAGAT |
