# Supplementary material for: Chilling injury of tomato fruit was alleviated under low-temperature storage by silencing Sly-miR171e with short tandem target mimic technology
Source: Front Nutr. 2022 Jul 25;9:906227. doi: 10.3389/fnut.2022.906227 (PMC9355414; doi:10.3389/fnut.2022.906227)
Supplement: Supplementary file 4 [file Image_1.pdf]

Fig. S1.The stably transformed miR171e-STTM line and miR171e-OE line were produced in the WT background by *A. tumefaciens* mediated genetic transformation. A total of 40 independent transgenic lines were detected in the T1 generation, including 23 STTM-171e lines and 17 miR171e-OE lines (Fig. S1 A and B). Similarly, 33 systems, including 16 STTM-171e lines and 17 miR171e-OE lines, were detected in the T2 generation (Fig. S1 C and D). Three STTM 171e and miR171e-OE lines were detected at T1 and T2. PCR amplification analysis of the presence of hygromycin (miR171e-STTM line) and kanamycin (miR171e-OE line) combined with agarose gel electrophoresis demonstrated that all three independent transgene lines were small inserted target genes (Fig. S1).

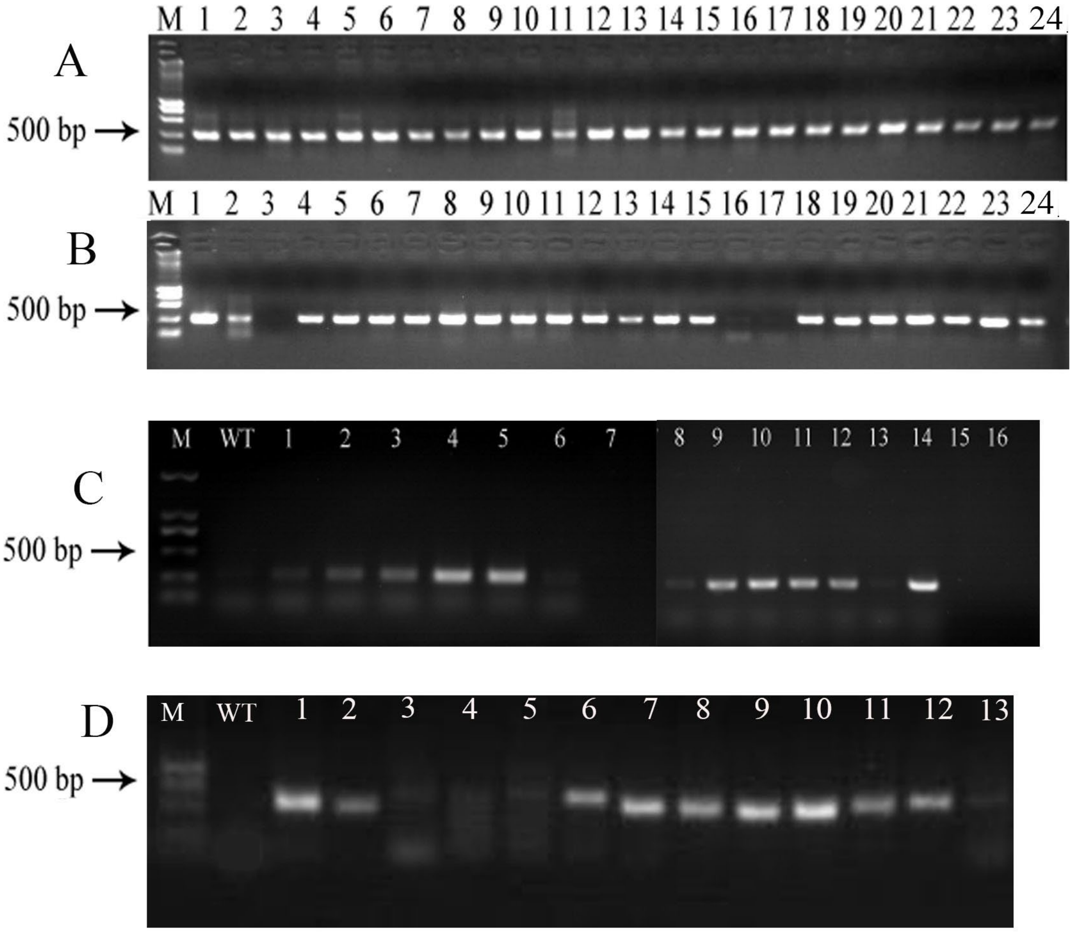
A
B
C
D
M
WT
500 bp
1 2 3 4 5 6 7 8 9 10 11 12 13 14 15 16 17 18 19 20 21 22 23 24
1 2 3 4 5 6 7 8 9 10 11 12 13 14 15 16 17 18 19 20 21 22 23 24
1 2 3 4 5 6 7 8 9 10 11 12 13 14 15 16
1 2 3 4 5 6 7 8 9 10 11 12 13
